# Measuring Experiential Avoidance and Posttraumatic Stress in Families

**DOI:** 10.3390/bs9100104

**Published:** 2019-09-27

**Authors:** Meaghan M. Lewis, Tamara M. Loverich

**Affiliations:** 1 Northwest MIRECC, VA Portland Health Care System, Portland, OR 97239, USA; 2 Psychology Department, Eastern Michigan University, Ypsilanti, MI 48197, USA; tpenix@emich.edu

**Keywords:** experiential avoidance, trauma, families, AAQ-II, MEAQ

## Abstract

Experiential avoidance (EA) is receiving attention as an emotion regulation strategy and critical factor in the development and maintenance of posttraumatic stress disorder (PTSD) Evidence suggests that EA explains co-varying relationships among topographically dissimilar problem behaviors. The transmission of emotion regulatory strategies is important to understanding the development of these problems. EA may be a learned response style. This conceptual framework was used to test parent EA as a predictor of young adult/older adolescent (offspring) EA, posttraumatic stress symptoms (PTSS), and problem behaviors in a university context as well as to test the best predictors of these outcomes individually for parents and offspring. Two measures of experiential avoidance, the unwillingness to be in contact with distressing emotions, thoughts, and memories were used to predict the outcomes of interest. The Acceptance and Action Questionnaire-II (AAQ-II) was the strongest and only statistically significant predictor of PTSS and problem behaviors for parents and offspring above and beyond trauma history, while the Multidimensional Experiential Avoidance Questionnaire (MEAQ) remained non-significant. Implications regarding measurement discrepancies, PTSS, and harmful behavior trajectories are discussed.

## 1. Introduction

Over half of the general population has experienced a potentially traumatic event (PTE) [1]. However, the lifetime prevalence rate for incidence of posttraumatic stress disorder (PTSD) is 6.8–7.8% [1,2]. Due to the striking disparity between trauma exposure and PTSD development, researchers have focused on the identification of psychological and behavioral risk factors that predict PTSD and how they develop. There has been a natural curiosity about familial transmission of risk and resilience factors.

At the heart of these inquiries is the study of emotion regulation in families. Emotion regulation strategies have been found to mediate the effect of trauma exposure on PTSD [3,4,5]. How people learn to relate to emotion appears to significantly impact their health. Functional contextual behaviorists conceptualize avoidance, a major symptom of PTSD, as a functional class of related behaviors called experiential avoidance (EA). EA refers to the unwillingness to withstand negatively appraised private events and subsequent efforts to escape or avoid such experiences [6]. The EA construct is a central process targeted in the Acceptance and Commitment Therapy (ACT) literature [7,8], and one that seems to have an important bearing on PTSD. 

The private events subsumed under the EA umbrella include thoughts, feelings, and memories, as well as behavioral predispositions and contexts engendering them [9]. Kashdan, Barrios, Forsyth, and Steger [10] describe EA as a generalized psychological vulnerability. In this line of thinking, there are certain circumstances in which EA can be understood as adaptive. These include instances in which EA is employed in a manner that still allows the pursuit of meaningful and valued goals. However, when utilized in a rigid and inflexible manner, EA is deemed pathogenic [11].

The body of research examining EA as a maladaptive regulatory style employed subsequent to trauma appears to be gradually aggregating. As EA is considered a transdiagnostic process [12], adopting this framework permits concentration on the functional processes involved in PTSD. There is evidence to suggest that EA predicts deleterious outcomes including PTSD symptom severity and general psychopathology [13], and aggressive behavior and trait anger beyond PTSD symptom severity [14]. Mediational analyses support the theory that EA mediates the effects of trauma exposure, including sexual victimization, on psychological distress and functioning in adulthood [15,16,17]. Chronic EA could conceivably limit psychological resilience. The problem behaviors some youth/young adults engage in could also be conceptualized as a form of hidden resilience [18]. While the problem behaviors may appear harmful, engaging in these behaviors could also lead to desired outcomes such as social acceptance and persist because they are reinforced in this way. The robust connection between EA and PTSD, which involves stressors by definition, supports such a conceptualization.

### 1.1. Behavioral Correlates of Avoidance 

Higher order factor analytic research has demonstrated that greater EA predicts excessive engagement in problem behaviors and relationships among topographically dissimilar problem behaviors [19]. Individuals may report engaging in multiple problem behaviors simultaneously. Kingston and colleagues’ results suggest that a number of these behaviors, regardless of form, could be the result of a unifying functional pathway. For example, research has shown that substance abusers are seven times more likely to develop a second addiction in comparison to non-abusers [20], while alcohol use has been found to be one factor that predicts internet overuse [21]. The relationship between problem behaviors and EA and dysfunctional emotion regulation strategies suggests that EA is a key mechanism through which various excessive problem behaviors emerge. Individuals who have an increased propensity to engage in EA may be more likely to engage in excessive problem behaviors in contexts that engender distressing private events such as exposure to PTEs. 

### 1.2. Family Context, Trauma, Avoidance, and Behavior Excesses 

One prominent finding throughout the emotion regulation literature is the importance of the family in modeling and teaching emotion regulation strategies [22,23], including EA. Parent psychopathology is a major risk factor that has been associated with poor emotion regulation strategies and problem behaviors among children [24,25]. The literature indicates that parent mental health status (predominantly maternal mental health) influences parenting trajectory, particularly if individuals do not seek mental health services [26]. Parents’ capabilities for managing distressing emotions appear to have an important influence on outcomes for children, adolescents, and young adults [27,28]. Children of parents who chronically mismanage distressing emotion in response to stressful life events may fail to acquire effective skills to manage emotion in response to their own distress. However, the harmful effects of trauma exposure on proclivity to engage in harmful behaviors are not well understood.

### 1.3. Rationale and Hypotheses 

In the developing body of research examining the interactions between parent and child emotion regulation processes, there is a need to more fully elucidate risk factors for the development of a maladaptive EA repertoire that may be utilized in response to traumatic stressors. We sought to integrate several areas of research that have not been investigated together. In this study, EA in parents was conceptualized as a vulnerability factor, impacting their responding during exposure to PTEs, and posttraumatic stress symptoms (PTSS) and problem behaviors. Parental modeling of EA and problem behaviors may then influence their child’s emotion regulation, resulting in an overgeneralized unwillingness to contact aversive private events. EA may increase the incidence of PTSS. Consequently, parent EA, PTSS, and problem behaviors may impact both offspring EA and related negative outcomes. The present study endeavored to extend the parent–child emotion regulation and trauma literature by analyzing these variables in older adolescents/young adults and their parents. The older adolescent/young adult participants were collapsed into one category labeled “offspring.” 

Given the significant influence of parents on their children’s learning and their shared environment and biology, we predicted significant associations between a parent’s EA, trauma history, PTSS, and problem behaviors and those of their offspring. We hypothesized that parent EA would predict parent PTSS and problem behaviors beyond parent trauma history. Offspring EA was likewise expected to predict offspring PTSS and problem behaviors beyond offspring trauma history. Lastly, parent EA was hypothesized to predict offspring EA beyond parent and offspring trauma history, parent problem behaviors, parent EA, and parent PTSS. All variables were expected to contribute unique variance to the predictive models, with EA emerging as the strongest predictor.

## 2. Materials and Methods

### 2.1. Participants 

The sample pool included a combination of 60 offspring and their 60 parents (*N* = 120). Participants were recruited through campus tour events and college classrooms at a Midwestern university. 

### 2.2. Procedure and Recruitment 

This study received Human Subjects Institutional Review Board approval prior to implementation. Trained research assistants approached potential participants who attended two university campus tour events for high school and transfer students interested in the university as well as the parent or primary caregiver. Attendance consisted of approximately a thousand individuals per respective event. Potential participants were orally informed of the opportunity to participate in an online survey regarding emotional responses to stressful events. They were provided with handouts about the study. Interested parents and offspring were escorted to a reserved computer laboratory. Informed consent and assent were provided online using the Survey Monkey website powered through the SONA system server. Participants were assigned confidential identification numbers with a shared number linking parent and offspring responses. Other potential participants were informed of the study through instructor oral recruitment in college classrooms. Instructors provided brief study information and explained the requirement of parent participation. College students were provided with the link to the SONA system survey containing an additional link to the online survey and confidential identification numbers were assigned. Parent participants were recruited through a question on the survey asking for the email or mailing address for the primary caregiver. They then received the study description, consent form, and link to the survey. All participants were compensated with gift cards in the amount of five dollars per respective participant. Extra credit was available at the discretion of course instructors for participants recruited from college classrooms. 

### 2.3. Measures 

After checking a box indicating consent or assent to participate, participants completed brief demographic information. A battery of self-report measures was then administered. 

Trauma exposure was assessed using the Life Events Checklist (LEC) [29], which is a 17-item checklist of trauma exposure used to assess exposure to PTEs (PTEs) [30]. The LEC inquires about PTE exposure such as natural disasters, assault with a weapon, sexual assault, transportation accidents, and any other type of stressful life experience. It allows participants to endorse the form of exposure (*happened, witnessed, learned about it, not sure, does not apply*) for each item. Individuals may endorse exposure to multiple PTEs. The LEC has adequate test–retest reliability over a seven-day period (*M* = 0.61) and good convergent validity with the Traumatic Life Events Questionnaire (TLEQ) (*M* = 0.70). 

Posttraumatic stress symptoms (PTSS) were evaluated using the PTSD Checklist-Civilian version (PCL-C) [30] which is a 17-item self-report measure assessing for the presence of criterion B, C, and D DSM-IV PTSD symptoms. It assesses levels of distress related to each symptom within the past thirty days. Items are rated on a scale 1 = *not at all* to 5 = *extremely.* A total severity score was calculated to determine symptom severity. Test–retest reliability for the PCL is reported at 0.92 (*p* < 0.001) among immediate retesters, 0.88 (*p* < 0.001) for participants within one-week retest intervals and 0.68 (*p* < 0.001) at two-week retest periods [31]. Cronbach’s alpha coefficients have demonstrated high internal consistency for the PCL-C total, re-experiencing, avoidance, and hyperarousal scales (0.94, 0.85, 0.85, 0.87 respectively). 

Experiential avoidance was measured using several self-report questionnaires. The Multidimensional Experiential Avoidance Questionnaire (MEAQ) [32] is a 62-item self-report measure that was developed to address issues with internal consistency and poor discriminant validity evidenced with other EA measures. The MEAQ contains questions pertaining to six dimensions of EA: behavioral avoidance, distress aversion, procrastination, distraction and suppression, repression and denial, and distress endurance. Items are rated on a Likert-type scale, ranging from 1 = *strongly disagree* to 6 = *strongly agree.* Higher scores are indicative of greater EA. The MEAQ has demonstrated good internal consistency and excellent convergent validity with avoidance measures and related constructs such as stress avoidance and alexithymia. It has excellent discriminative validity and broad assessment of unique content through the six subscales. In the initial validation study, the MEAQ total internal consistency was excellent (α = 0.92) with the subscales averaging good internal consistency across samples (α = 0.83). The MEAQ has not yet been widely used in EA research.

The Acceptance and Action Questionnaire-II (AAQ-II) [9] is a 7-item self-report measure of EA, rated on a seven-point Likert-type scale (ranging from 1 = *never true* to 7 = *always true*) with higher scores suggesting increased EA and lower scores reflecting greater psychological flexibility. The AAQ-II has demonstrated good internal consistency (alpha coefficient mean of 0.84). It has good test–retest reliability of 0.81 and 0.79 for twelve and three months, respectively. It is the most widely used measure of EA. There is some discussion in the literature regarding the extent to which the AAQ-II’s measurement of experiential avoidance is unique or overlaps with factors such as neuroticism, negative affect, and psychological distress [33,34]. Given these critiques, we were interested in comparing the AAQ-II and MEAQ as predictors of PTSS and problem behaviors. 

The propensity to engage in problematic behaviors was assessed using the Composite Measure of Problem Behaviors (CMPB) [35], which is a 46-item measure of ten different problems behaviors: nicotine use, deliberate self-harm, excessive internet/computer game use, drug use, excessive exercise, excessive alcohol use, binge eating, sexual promiscuity, aggression, and restrictive eating. Participants endorse the items on a six-point scale (ranging from 1 = *very unlike me* to 6 = *very like me*). Confirmatory factor analysis delineated a common higher order factor explaining co-variation between the subscales, supporting the hypothesis that such behaviors may be explained by a common functional pathway. The subscales have good construct validity with other psychometrically validated measures. The CMPB also has good internal consistency (α = 0.73–0.91) and test–retest reliability (95% CI). Reliability estimates were stable across time periods of: two weeks (*r* = 0.73–0.98), two-four months (*r* = 0.69–0.91), and eight-fourteen months (*r* = 0.65–0.91).

## 3. Results

### 3.1. Analytic Strategy 

Statistical Packaging for the Social Sciences (SPSS) was used to analyze data. Little’s Missing Completely at Random (MCAR) test was conducted to test for non-random patterns of missing data and was non-significant, indicating that the data were missing in a random pattern. Thus, data were considered MCAR and missing data were addressed using the expectation maximization approach. Bivariate associations were assessed using Pearson product moment correlation analyses. Correlations were computed for parents and offspring independently and then together to determine the relationships between parent and offspring variables. Hierarchical multiple regression analyses were used to predict outcomes including PTSS and problem behaviors from AAQ-II scores, MEAQ scores, and exposure to trauma. To correct for positive skewness of the distribution for the PCL-C in offspring, a logarithm + 10 was computed. No further transformations were warranted.

### 3.2. Descriptive Statistics 

Participants consisted of sixty parent and offspring dyads (*N* = 120). Forty-eight parent and offspring pairs (*n* = 96) were recruited from campus tour events, and 12 (*n* = 24) were recruited from college classrooms. The mean age reported by offspring was 18 (*SD* = 3), with a range of 15–31. Parent participants reported a mean age of 46 (*SD* = 7.8), ranging in age from 32–70. The majority of parents and offspring were female (75% and 73%, respectively). Thirty-two percent and 33% of parents and offspring respectively identified as other than European-American. Ninety-eight percent of parents and 83% of offspring reported exposure to at least one PTE. The most common PTE reported by parents was a transportation accident, while offspring rated “other stressful life event or experience” as their most common PTE. Parents and offspring most often rated the “sudden, unexpected death of someone close to you” as their most distressing PTE. In terms of severity of PTSS, the sample fell in the mild symptomatic range. Bond et al. [9] considers this threshold for clinically distressed on the AAQ-II. 

Bivariate correlations and descriptive statistics are presented in Table 1 by group. Table 2 displays the relationships between parent and offspring variables. A strong positive correlation emerged between parent AAQ-II scores and problem behaviors as well as their MEAQ scores and problem behaviors. Parent PTSS were strongly correlated with their AAQ-II and MEAQ scores. Parent PTSS were also strongly linked with parent problem behaviors. Offspring AAQ-II scores were strongly associated with their overall engagement in problem behaviors, a discrepant finding from the non-significant association between MEAQ scores and problem behaviors. Offspring PTSS were strongly linked with offspring AAQ-II scores, but not MEAQ scores. Finally, offspring PTSS were strongly associated with their problem behaviors. 

As seen in Table 2, AAQ-II scores were associated in parents and offspring (*r* = 0.27, *p* < 0.05). Contrary to our prediction, MEAQ scores were not significantly related for parents and offspring. Total problem behaviors, PTSS, and trauma exposure were also correlated for the pairs. In evaluating individual subscales of the CMPB, it was noted that parent and offspring deliberate self-harm (*r* = 0.31, *p* < 0.05), aggression (*r* = 0.31, *p* < 0.05), and internet overuse (*r* = 0.29, *p* < 0.05) were significantly correlated. 

We also evaluated for potential relationships between parent and offspring MEAQ subscale scores and AAQ-II scores at the bivariate level. Similar to findings at the total score level for the MEAQ scores, we did not find a significant relationship between any subscale between parents and their offspring. However, we did find a significant association between parent procrastination and offspring AAQ-II scores (*r* = 0.27, *p* < 0.05). These results can be found in Table 3. We also investigated for potential gender differences in EA, PTSS, and problem behaviors for both parents and offspring through conducting a series of independent samples *t*-tests. No significant differences were found between means for any of these variables as a function of gender for both parents and offspring separately. However, we did find a significant difference in means between male and female parents such that females were more likely to have offspring who engaged in more problem behaviors, *t*_(58)_ = −2.293, *p* < 0.05. Given the sample size difference between parental gender (*n* = 44 female, *n* = 16 male), it is likely that these differences can be attributed to the sample consisting predominately of women. Because of this, no further analyses were pursued related to gender differences.

### 3.3. Hierarchical Multiple Regression Analyses 

Hierarchical multiple linear regression analyses were conducted to elucidate which variables most strongly predicted parent PTSS and engagement in problem behaviors. Parallel regression models were calculated for offspring. Regression analyses with parent EA, PTEs, PTSS, and problem behaviors as predictors of offspring EA, PTSS, and problem behaviors were conducted. Although parent MEAQ and AAQ-II scores were strongly correlated (*r* = 0.60, *p* < 0.01), these associations and relevant others did not reach the 0.80 criterion for multicollinearity.

The first regression equation was employed to determine the amount of variance parent trauma history and parent EA contributed to parent PTSS. Parent trauma history was entered into the model first. Parent EA as assessed separately by the AAQ-II and MEAQ was entered in the subsequent block. Parent trauma history explained 3.7% of the variance in parent PTSS, *R²* = 0.037, *F* (1, 58) = 2.247, *p* = 0.139. As such, it did not make a significant unique contribution to parent PTSS. The addition of parent EA as indicated by AAQ-II and MEAQ scores explained 46.3% of the variance in parent PTSS. Parent EA contributed an additional 42.6% of the variance in parent PTSS, *R²* Δ = 0.426, *F* (2, 56) = 16.091, *p* = 0.001 beyond parent trauma history. Parent AAQ-II scores made a unique contribution to the prediction of parent PTSS (*β* = 0.572, *t* = 4.660, *p* < 0.001); however, parent MEAQ scores did not. Partial support was thus gathered to support this hypothesis regarding the AAQ-II, but not the MEAQ. Findings are summarized in Table 4. As a result, a subsequent regression equation in which the six subscales of the MEAQ were included to assess potential incremental contributions of different forms of avoidance was computed. Parent AAQ-II symptoms remained the only significant predictor in this model as well and thus subsequent related analyses were not pursued.

Potential unique contributions of parent trauma history and parent EA to prediction of parent problem behaviors were then assessed. Parent trauma history was entered in block one, while parent EA (AAQ-II and MEAQ) was entered in block two. Parent trauma history alone explained 4.9% of the variance in parent problem behaviors, *R²* = 0.049, *F* (1, 58) = 3.019, *p* = 0.088. The addition of parent EA in block two contributed 46.3% of the variance in parent problem behaviors and an additional 42.6% of the variance beyond parent trauma history, *R²* Δ = 0.463, *F* (2, 56) = 15.866, *p* = 0.001. Parent AAQ-II scores were the strongest, and only, statistically significant predictor of parent problem behaviors (*β* = 0.560, *t* = 4.29, *p* < 0.001). Findings demonstrate partial support of initial hypotheses. These findings are displayed in Table 5. 

Offspring trauma history was entered in the initial block of the next regression equation, while offspring EA scores were entered in block two. Offspring trauma history explained 3% of the variance in PTSS, *R² =* 0.03, *F* (1, 58) = 1.784, *p* = 0.187. Entering EA into the equation accounted for 37% of the variance in PTSS, and 34% additive variance beyond trauma history, *R²* Δ = 0.340, *F* (2, 56) = 15.866, *p* = 0.001. In the total model, EA as assessed by the AAQ-II demonstrated the highest, and only, statistically significant beta value (*β* = 0.618, *t* = 5.278, *p* < 0.001). Findings suggest that the EA construct measured using the AAQ-II was the strongest predictor of PTSS. These results provide partial support for the initial hypothesis as EA was the strongest predictor, but only as assessed by the AAQ-II. A summary of this regression analysis is displayed in Table 6. 

Predictors of offspring problem behaviors were then evaluated. Offspring trauma history was entered in block one and offspring EA scores were input in block two. Offspring trauma history did not significantly contribute to offspring problem behaviors, *R²* = 0.004, *F* (1, 58) = 0.219, *p* = 0.642. At block two, the addition of EA accounted for 26% of the variance in offspring problem behaviors and accounted for 25.7% of the variance beyond trauma history, *R²* Δ = 0.257, *F* (2, 56) = 6.584, *p* = 0.001. Only AAQ-II scores showed a unique contribution to offspring problem behaviors (*β* = 0.559, *t* = 4.411, *p* < 0.001). Partial support was thus provided for the hypothesis that offspring EA most strongly predicts engagement in problem behaviors. Discrepancies were again observed in measurement of EA. Model coefficients are displayed in Table 7. 

Hierarchical multiple regression analyses were also used to assess parent trauma history, EA, PTSS, and problem behaviors as predictors of offspring EA, PTSS, and problem behaviors. It was hypothesized that parent EA would be the strongest predictor of offspring EA. To test these hypotheses, an initial hierarchical multiple regression model was computed with offspring EA (AAQ-II) as the outcome variable. The predictors in the model were parent and offspring trauma history in block one, parent PTSS in block two, parent problem behaviors in block three, and parent EA (both AAQ-II and MEAQ) in block four. This model did not account for a statistically significant amount of the variance in offspring EA. Hence, unique contributions of the predictors were not assessed. A separate regression equation was conducted using these same variables predicting offspring MEAQ scores. The model was non-significant as well. A third regression equation was estimated with parent trauma history at block one and parent AAQ-II scores in block two, with offspring AAQ-II scores serving as the criterion. In this model, parent trauma history explained 2.4% of the variance, *R²* = 0.024, *F* (1, 58) = 1.407, *p* = 0.240 in block one, while the addition of parent EA contributed 6% of the variance beyond parent trauma history, *R²* Δ = 0.071, *F* (1, 57) = 2.981, *p* = 0.039. In this final model, parent EA accounted for unique variance in offspring EA as measured by the AAQ-II (*β* = 0.267, *t* = 2.115, *p* < 0.05). Although parent EA was found to be a unique contributor in the subsequent regression, the hypothesis that it would strengthen the predictive model above parent and offspring trauma history, parent PTSS, and parent problem behaviors was not supported. Results are presented in Table 8.

The final hypothesis was tested by entering parent and offspring trauma history in the first block of a hierarchical multiple regression. Parent PTSS were entered in the subsequent block. Parent problem behaviors were added in block three, while parent EA was entered in the fourth block. These variables were estimated as predictors of offspring PTSS. At block one, parent and offspring trauma history explained 6% of the variance in older adolescent PTSS, *R²* = 0.064, *F* (2, 57) = 1.953, *p* = 0.151. In block two, adding parent PTSS contributed 14.7% of the variance and 8.3% beyond trauma history in block one, *R²* Δ = 0.083, *F* (1, 56) = 5.426, *p* = 0.023. Parent problem behaviors explained no additional variance in block three, *R²* Δ = 0.000, *F* (1, 55) = 0.028, *p* = 0.867. In block four, the addition of parent EA contributed only 15.7% of the total variance in offspring PTSS, and thus did not make a significant contribution beyond that of the other predictor variables. In the final model, parent PTSS were the best predictors of offspring PTSS (*β* = 0.300, *t* = 2.329, *p* < 0.05). These coefficients are displayed in Table 9.

In a subsequent regression equation for offspring problem behaviors, parent and offspring trauma histories were entered in the initial block. Parent PTSS were entered in block two. Parent problem behaviors were then added to the model in block three. Finally, parent EA was added in block four. The hypothesis was not supported as the model did not account for a significant amount of the variance in offspring problem behaviors. As we were also interested in determining if individual MEAQ subscales contributed independently to the prediction of PTSS and problem behaviors, we re-ran all regression equations with the inclusion of MEAQ subscales as opposed to the MEAQ total score. However, results remained inconsistent and for the purposes of readability, only the regression models with MEAQ total scores are displayed.

## 4. Discussion 

The aims of this study were to improve understandings of specific vulnerability factors for EA, PTSS, and engagement in overt, problematic behaviors posttrauma, and their development in families. Problem behaviors were conceptualized based on research findings that such behavior may serve an EA or avoidant coping function [19]. Study design was informed by an intergenerational perspective in which parental avoidance strategies, traumatic events, and distress in the form of PTSS were analyzed for their effects on their offspring. Relative contributions of individual trauma history and EA on important outcomes including PTSS, EA and problem behaviors were also addressed. 

Participants were predominately Caucasian and the majority of both parents and offspring described themselves as female. Almost every participant reported exposure to at least one PTE. Both the parent and offspring groups endorsed mild PTSS. Contrary to hypotheses, trauma exposure was not associated with PTSS within and between family members. It is possible that some symptoms participants endorsed on the PCL-C (e.g., difficulties concentrating or falling asleep) were not trauma related. No clinical interviews were administered and therefore explicit speculations cannot be formed. The absence of a correlation between trauma history and PTSS may be due to statistical power or sample characteristics. It is worth noting that this was a college-seeking sample that may already be demonstrating resilience which may in part be due to having more access to resources (i.e., financial resources, social support) that may increase one’s psychological flexibility. While individuals who do not pursue higher education are not necessarily less resilient, their decision to not pursue college could be due to lower access to resources. A clinical sample might have more significant associations between trauma history and PTSS. At the same time, these findings bolster other studies that demonstrate that PTSS are not a given in the wake of a PTE.

Parent and offspring trauma histories were correlated, and the influence of shared events could be one potential explanation for this relationship. The number of individuals reporting exposure to “sudden violent death,” and “serious injury, harm, or death you caused to someone else” corresponded for some family members (*n* = 4), but other events were more divergent. The event endorsed as most distressing (“sudden unexpected death of someone close to you”) was common among parents and offspring. Parent and offspring PTSS were also associated. This finding seems consistent with other results in the parent–child posttraumatic stress/PTSD literature [27]. One potential interpretation of the link between PTSS in parents and offspring could be that posttraumatic stress responses are modeled by parents. Symptom trajectory may also be explained by independent responses to parallel, stressful events. It also seems possible that parental PTSS are experienced as a stressor or perhaps even a PTE for their children. Another important finding was that offspring had higher mean scores on all measures than parents did. In addition to parents modeling poor coping strategies, their offspring may also be more likely to be exposed to trauma early. From a behavioral perspective, emotion regulation strategies are shaped through trials of reinforcement over time. Therefore, it also seems likely that offspring reported higher PTSS, EA, and problem behaviors because they are less practiced at regulating emotions and thus experience more discomfort earlier in life. The nature of the current stressors in their lives also may vary as a function of where they are at developmentally. 

It is notable that a strong relationship was observed between AAQ-II scores and PTSS for both groups, but that MEAQ scores only linked with PTSS for parent participants (*r* = 0.47, *p* < 0.01) while there was not a significant relationship between MEAQ scores and PTSS for offspring. As the MEAQ is a measure of specific avoidance strategies, perhaps parents are more practiced at certain avoidance strategies which have been shaped over time. It would be interesting to study the development of these strategies longitudinally to further support such a trajectory. AAQ-II scores have been found to be robust predictors of PTSS among different groups including convenience and clinical samples, and military veterans [15,36,37]. However, the MEAQ’s relationship to PTSD has been examined in only one published study to the authors’ knowledge [38], finding that pathways to alcohol use and alcohol use consequences differed between individuals with high and low levels of PTSD symptoms, and noting links between the distress aversion MEAQ subscale and alcohol-related consequences through social motives. Our study is unique in its use of both the MEAQ and AAQ-II as measures of EA. While the measures are related in the expected direction, there appear to be significant differences in either the construct(s) they are measuring or how they are measuring it/them, an issue discussed in greater detail below. 

One possibility that must be considered is that the correlation between AAQ-II scores and PTSS is due to content overlap. For example, the items “my painful experiences and memories make it difficult for me to live a life I would value” and, “my painful memories prevent me from living a fulfilling life” seem reflected in the construct of posttraumatic stress. Some items may be indicating a very similar, if not the same, construct. The MEAQ includes comparable items (e.g., “when upsetting memories come up, I try to think of other things”), but the multifactorial nature of the broader measure may leave room for aspects of EA that do not overlap so significantly with posttraumatic stress. Because there were not significant findings regarding the relationship between any parent and offspring MEAQ subscales or total scores, there is stronger evidence that the MEAQ is measuring a different construct than the AAQ-II. If the AAQ-II is indeed a better measure of negative affect intensity than EA, our results then suggest that parents with greater negative emotionality tend to model this for their children. If the MEAQ is a more accurate measure of EA, then our results indicate that EA was not necessarily modeled for children. Instead, an EA repertoire may be learned through exposure to other environmental consequences beyond one’s immediate family. However, replicating these results with a second parent and clinical sample would bolster this idea. 

Divergent findings on the AAQ-II and MEAQ for parents and offspring may alternatively reflect the developmental trajectory of EA. It is possible that early in life one may learn an unwillingness to experience painful thoughts and feelings, but the behaviors one enacts to be less in contact with those thoughts and feelings mature over time. Thus, the AAQ-II total score and MEAQ distress aversion subscale may capture the non-accepting stance toward private events, while the other subscales of the MEAQ reflect growing skillfulness in avoidance. This seems supported by the similarity in scores for parents and offspring on the distress aversion subscale (*M* = 43; *M* = 40, respectively for offspring and parents), which did not differ statistically. It could also be theorized that, given the criticisms of the AAQ-II’s internal consistency [33,34], what was actually captured was a relationship between neuroticism/negative affect in parents and their offspring. 

Parent and offspring AAQ-II scores were associated with one another, while there was no significant link between their MEAQ scores. This suggests that the unwilling stance toward emotion is detectable in adolescence/young adulthood, while the external avoidant behaviors develop later in the lifespan. To bolster this notion, elevated use of problem behaviors linked with both measures of EA for parents than for offspring. Earlier studies of EA and PTSS have utilized mostly adult samples, possibly obscuring this finding. As such, additional investigation of this hypothesis is needed.

Another related possibility is that EA and PTSS may become more elevated in parents due to relatively greater environmental demands such as supporting a family. Length of time relying on EA may also be a critical variable. Parents may acquire a stronger history of reinforcement for engaging in EA and hold significantly different and more developed repertoires than their offspring. Exposure to increasingly difficult or stressful life events as time evolves may interact with already shaped or evolving EA repertoires. 

A strong relationship was elucidated between AAQ-II scores and problem behaviors for both parents and offspring. However, no significant association emerged between MEAQ scores and problem behaviors for offspring. This is perplexing given that, conceptually, the MEAQ subscales of behavioral avoidance, procrastination, distraction and suppression, and distress endurance appear related to problematic behaviors such as substance abuse, binge eating and others. This finding may support our idea that the MEAQ may be tapping into more adult methods of experientially avoiding, also supported by data that demonstrated that more problem behaviors linked with both measures of EA for parents than for their offspring. There was also no significant relationship between trauma exposure and engagement in problem behaviors, a finding contrary to that of the Kingston, Clarke and Remington [19] and Polusny et al. [16] studies. Total CMPB scores were associated in parents and offspring; however, parent and offspring deliberate self-harm, aggression, and internet overuse were more strongly associated and it would seem that these problem behaviors are more salient for an adolescent/young adult population. Parents’ CMPB score means differed significantly from offspring CMPB score means, *t*_(59)_ = 3.256, *p* = 0.002, supporting the idea that such behavior is shaped across time. Because the relationship between parent MEAQ scores and problem behaviors was significant, it seems that the relationship between specific avoidance strategies and problem behaviors requires a longer learning history to condition. Importantly, problem behaviors may also serve a different function in younger populations. Many of the problem behaviors assessed on the MEAQ (i.e., substance use) may initially begin because they serve a positive reinforcement function and result in increased access to other positive reinforcers such as peer support. However, over time their function may alter as, for example, dependence on substances strengthens or alternatively if exposure to negative life events (i.e., traumatic stressors) increases in frequency. 

### Experiential Avoidance Measurement: Understanding Avoidance in Families 

The significant discrepancies in measurement of EA with the AAQ-II and MEAQ are interesting. From a basic behavioral perspective, the two-factor theory of avoidance involves both a respondent conditioning component (direct exposure to aversive private events) as well as an operant component, including direct attempts to alter contact with aversive private events. Although one of the main criticisms to the two-factor theory of avoidance includes the idea that many individuals avoid aversive stimuli that they have not directly encountered, the key factor that discriminates EA theoretically is that escape and avoidance behavior become chronic and overgeneralized across contexts. 

Gámez and colleagues [32] argued that the AAQ and AAQ-II items assess dysfunctional distress and psychological inflexibility that fall beyond the scope of the EA construct. Reading the items of the AAQ-II, we would argue that it is primarily a measure of evaluation of, and unwillingness to experience negatively evaluated, private events (thoughts, feelings and sensations). This is the necessary feature of the EA construct. We assert that behaviors that are enacted to escape or avoid contact with private events are not the same as the unwilling stance itself. Many of the behaviors that function to escape/avoid the unwanted thoughts or feelings may not be available to all people or certain groups of people, and those behaviors may serve other functions as well. They are not equivalent to the stance itself. They are the second factor in the two-factor theory. This may be the fundamental difference between the AAQ-II and the MEAQ. The MEAQ includes both the stance and the behaviors to escape and avoid being in contact with private events. As such, it appears more likely to reflect developmentally more advanced behavioral repertoires associated with EA, and to be subject to the endorsement of behaviors that are excessive in nature, but that do not serve an EA function. It is a less distilled measure of EA. Future researchers interested in measuring more specific avoidance strategies (i.e., behavioral avoidance, procrastination) and the specific behaviors individuals engage in to avoid (i.e., alcohol/drug use) may select the MEAQ. However, if measuring EA related to internalizing symptoms such as depression, anxiety, and negative emotionality, perhaps the AAQ-II may be a better measure. 

Offspring and parent AAQ-II scores were the strongest predictors of their own respective PTSS and problem behaviors. However, in a separate model parent PTSS were the strongest predictor of offspring PTSS, beyond parent and offspring trauma history, parent problem behaviors, and parent EA. Polusny and colleagues [28] also found that parent PTSD predicted offspring PTSD beyond parent EA. Although it was hypothesized that parent PTSS would contribute unique variance to the prediction of offspring PTSS, initially it was presumed that parent EA would be the most robust predictor. These particular findings suggest that parental PTSS may be a stronger risk factor for offspring PTSS than parent EA. In a model removing offspring trauma history, offspring EA, and parent problem behaviors, parent AAQ-II scores predicted offspring EA beyond parent trauma history. This model, though significant, was not as strong as expected. As data were not collected from both parents, a complete account of parental EA could not be included but will be important in future studies. These results seem to illustrate that how parents manage their own affect matters to their children’s wellbeing. Parent EA is a risk factor for offspring EA, although it is not the only factor to consider. Taken as a whole, EA may be the key mechanism in understanding individual relationships, while family contextual trauma sequelae trajectories are likely complicated by the influence of multiple factors. 

The model predicting offspring problem behaviors was non-significant, while parent AAQ-II scores were important predictors of their engagement in problem behaviors. Implications regarding the function of the behavior of interest may help understand this discrepancy. Engagement in problem behaviors may initiate in the service of negative reinforcement of unwanted private events and may be associated with positive reinforcers such as increased social interaction. Over time, continued use of these behaviors could result in harmful consequences. Problem behaviors may then be employed as a pervasive pattern of overgeneralized negative reinforcement.

## 5. Limitations and Future Research 

Limitations foremost include reliance on a cross-sectional design and use of self-report instruments to retrospectively assess the variables of interest. The use of a hybrid convenience and community sample may also carry sample characteristics that diverge from a more general community or clinical sample, thereby limiting the generalizability of our findings. It will be important for future studies to attempt to replicate these results using other samples. Differences between measures of EA provide fruitful evidence that behavioral measures of EA are necessary. Observed measurement discrepancies provide support for a need to validate both overt non-verbal behavioral measures as well as improved self-report measures of EA such as Ecological Momentary Assessments. 

The limited sample size prohibited use of more sophisticated modeling techniques. Paired analyses (intraclass correlation coefficients, pooled regression) were not conducted as there was no clear independent variable hypothesized to mutually impact parents and offspring (e.g., effect of divorce on two partners). Future studies could include larger samples to allow for more advanced estimation of factors including mediating and moderating variables. Longitudinal investigations are also necessitated in future research, particularly given recent findings that have supported the trait-like nature of EA [39]. Given our results, we noted higher means on both the MEAQ and AAQ-II for the younger portion of our sample. Yet, because there was only a relationship with MEAQ scores and problem behaviors for parent participants, we believe certain EA strategies may become more trait-like as the lifespan progresses. There may be new implications for the role of EA in acute events such as PTEs given that EA is seen as enduring yet its inverse may be trained through acceptance-based therapy protocols. It should also be noted that some avoidant tendencies (i.e., behavioral inhibition/avoidance) could be heritable to a degree and thus a behavioral conceptualization may be incomplete. 

Another limitation concerns our method. Most parents and offspring completed the measures in a university setting and were within proximal distance of one another. Expectancy biases and the concern that loved ones may see their responses could have influenced willingness to honestly self-report difficult life experiences, avoidance strategies, as well as distress and problem behaviors. EA is thought to comprise a behavioral unwillingness to be present with distressing private experiences [8]. Thus, individuals who rely most strongly on this response style may have opted not to participate. Perhaps more importantly, self-reporting on one’s own EA may require a degree of awareness with the present moment that seems incongruent to the construct of interest [40]. Responses were also drawn from one parent and one offspring, limiting consideration of multiple parents and offspring. A more involved study could examine individual difference characteristics incorporating both parents, parents with different levels of EA, or account for non-traditional family influences. Given the nature of our recruitment strategy, we did include offspring who live at home and who do not live at home anymore. It is likely that individuals who still reside with their parents are more strongly impacted by the EA strategies of their parents than those who do not. 

## 6. Conclusions 

The present study collected data from a sample of parents and offspring to further understand transmission of EA posttrauma and propensity to develop psychological symptoms and engage in harmful behaviors. Findings illustrate the utility of one empirically validated measure of EA, the AAQ-II, in predicting PTSS and problem behaviors independently in parents and offspring, while the MEAQ did not predict these outcomes. EA or general psychological inflexibility appears to operate as a risk factor for harmful sequelae experienced in families. Specific avoidance strategies may hold idiographic meaning to predicting harmful outcomes. Parent PTSS were the strongest predictors of offspring PTSS, replicating empirical findings from the literature [27]. Given these findings and the success of many protocols in reducing EA, PTSS, and problem behaviors, the outlook is hopeful for parents who would like to prevent negative outcomes for themselves and their children. However, it is important to note that pathways to problem behaviors and PTSS are likely complex and not fully explained by family context. 

## Figures and Tables

**Table 1:** Means, standard deviations, and bivariate correlations of study variables by group.

Variable	*M*	*SD*	1	2	3	4
**Parent variables**						
1. EA—MEAQ	180	34	-			
2. EA—AAQ-II	17	9	0.60 **	-		
3. PTSS	30	13	0.47 **	0.65 **	-	
4. Problem behaviors	100	22	0.38 **	0.59 **	0.59 **	-
**Offspring variables**						
1. EA—MEAQ	202	32	-			
2. EA—AAQ-II	24	9	0.45 **	-		
3. PTSS	38	13	0.24	0.66 **	-	
4. Problem behaviors	111	24	0.03	0.43 **	0.54 **	-

*Note. N* = 60 parents and 60 offspring, ** *p* < 0.01.

**Table 2:** Bivariate correlations of study variables between parents and offspring.

Parents	Offspring						
Variable	1	2	3	4	5	6	7
1. EA—MEAQ	−0.08	-					
2. EA—AAQ-II	0.01	0.27 *	-				
3. PTSS	0.04	0.13	0.26 *	-			
4. Problem behaviors	−0.20	0.15	0.20	0.26 *	-		
5. Deliberate self-harm	−0.04	0.22	0.13	0.33 **	0.31 *	-	
6. Internet overuse	0.01	0.23	0.08	0.05	0.16	0.29 *	
7. Aggression	−0.11	0.17	0.10	0.23	−0.04	0.26 *	0.31 *

*Note. N* = 60 parents and 60 offspring, * *p* < 0.05, ** *p* < 0.01.

**Table 3:** Bivariate correlations of parent and offspring EA.

	Offspring MEAQ Subscales						Offspring AAQ-II
Parent MEAQ Subscales	1	2	3	4	5	6	7
1. Behavioral Avoidance	0.03	−0.02	−0.07	−0.03	−0.11	0.19	0.01
2. Distress Aversion	0.23	0.15	−0.16	0.02	−0.14	0.18	0.07
3. Procrastination	−0.08	−0.12	0.24	−0.11	−0.12	−0.04	0.26 *
4. Distraction and Suppression	0.11	0.01	−0.09	−0.10	−0.03	0.15	−0.07
5. Repression and Denial	−0.15	−0.08	−0.07	0.00	0.12	0.07	−0.09
6. Distress Endurance	0.08	−0.00	−0.03	−0.02	0.16	−0.06	0.01
7. Parent AAQ-II	0.08	0.07	0.09	0.01	−0.12	0.07	0.27 *

*Note. N* = 60 parents and 60 offspring, * p < 0.05.

**Table 4:** Summary of hierarchical linear regression predicting parent PTSS.

Block	Variable	*B*	*SE B*	*β*	*t*	*R²*	*R² Δ*	*F*
1	Parent trauma exposure					0.037	0.037	2.247
Measure	LEC	0.013	0.009	0.193	1.499			
2	Parent EA					0.463	0.426	16.091 ***
Measure	LEC	0.011	0.006	0.166	1.688			
	AAQ-II	0.010	0.002	0.572	4.660 ***			
	MEAQ	0.001	0.001	0.122	0.997			

*Note.* *** *p* < 0.001.

**Table 5:** Summary of hierarchical linear regression predicting parent problem behaviors.

Block	Variable	*B*	*SE B*	*β*	*t*	*R²*	*R² Δ*	*F*
1	Parent trauma exposure					0.049	0.049	3.019
Measure	LEC	2.073	1.193	0.222	1.737			
2	Parent EA					0.463	0.426	15.866 ***
Measure	LEC	1.851	0.971	0.199	1.906			
	AAQ-II	1.344	0.313	0.560	4.290 ***			
	MEAQ	0.029	0.086	0.043	0.333			

*Note.* *** *p* < 0.001.

**Table 6:** Summary of hierarchical linear regression predicting offspring PTSS.

Block	Variable	*B*	*SE B*	*Β*	*t*	*R²*	*R² Δ*	*F*
1	Offspring trauma exposure					0.030	0.030	1.784
Measure	LEC	0.013	0.010	0.173	1.336			
2	Offspring EA					0.370	0.340	15.866 ***
Measure	LEC	0.012	0.008	0.152	1.435			
	AAQ-II	0.010	0.002	0.618	5.278 ***			
	MEAQ	0.000	0.001	−0.097	−0.825			

*Note.* *** *p* < 0.001.

**Table 7:** Summary of hierarchical linear regression predicting offspring problem behaviors.

Block	Variable	*B*	*SE B*	*β*	*t*	*R²*	*R² Δ*	*F*
1	Offspring trauma exposure					0.004	0.004	0.219
Measure	LEC	0.738	1.577	0.061	0.468			
2	Offspring EA					0.261	0.257	6.584 ***
Measure	LEC	0.579	1.384	0.048	0.418			
	MEAQ	−0.162	0.091	−0.225	−1.771			
	AAQ-II	1.423	0.323	0.559	4.411 ***			

*Note.* *** *p* < 0.001.

**Table 8:** Summary of hierarchical linear regression predicting offspring experiential avoidance.

Block	Variable	*B*	*SE B*	*β*	*t*	*R²*	*R² Δ*	*F*
1	Parent trauma exposure					0.024	0.024	1.407
Measure	LEC	0.596	0.502	0.154	1.186			
2	Offspring EA					0.095	0.071	2.981 *
Measure	LEC	0.555	0.488	0.143	1.137			
	AAQ-II	0.266	0.126	0.267	2.115 *			

*Note.* * *p* < 0.05, criterion variable assessed using AAQ-II.

**Table 9:** Summary of hierarchical linear regression predicting offspring PTSS from parent variables.

Block	Variable	*B*	*SE B*	*β*	*t*	*R²*	*R² Δ*	*F*
1	Parent trauma exposure	0.668	0.757	0.119	0.883	0.064	0.064	1.953
	Offspring trauma history	1.310	0.925	0.191	1.417			
2	Parent PTSS	0.315	0.135	0.300	2.329 *	0.147	0.083	3.212 *
3	Parent problem behaviors	0.016	0.095	0.026	0.168	0.147	0.000	0.028
4	Parent EA							
Measure	AAQ-II	0.168	0.289	0.116	0.582			
	MEAQ	0.017	0.064	0.043	0.266			

*Note.* * *p* < 0.05.
